# Shared Biological Pathways Between Alzheimer’s Disease and Ischemic Stroke

**DOI:** 10.3389/fnins.2018.00605

**Published:** 2018-09-07

**Authors:** Pan Cui, Xiaofeng Ma, He Li, Wenjing Lang, Junwei Hao

**Affiliations:** ^1^Department of Neurology, Tianjin Neurological Institute, Tianjin Medical University General Hospital, Tianjin, China; ^2^Key Laboratory of Post-neurotrauma Neuro-repair and Regeneration in Central Nervous System, Tianjin Neurological Institute, Ministry of Education and Tianjin City, Tianjin, China

**Keywords:** Alzheimer’s disease, ischemic stroke, genome-wide association studies, gene-based analysis, pathway-based analysis

## Abstract

Alzheimer’s disease (AD) and ischemic stroke (IS) are an immense socioeconomic burden worldwide. There is a possibility that shared genetic factors lead to their links at epidemiological and pathophysiological levels. Although recent genome-wide association studies (GWAS) have provided profound insights into the genetics of AD and IS, no shared genetic variants have been identified to date. This prompted us to initiate this study, which sought to identify shared pathways linking AD and IS. We took advantage of large-scale GWAS summary data of AD (17,008 AD cases and 37,154 controls) and IS (10,307 cases and 19,326 controls) to conduct pathway analyses using genetic pathways from multiple well-studied databases, including GO, KEGG, PANTHER, Reactome, and Wikipathways. Collectively, we discovered that AD and IS shared 179 GO categories (56 biological processes, 95 cellular components, and 28 molecular functions); and the following pathways: six KEGG pathways; two PANTHER pathways; four Reactome pathways; and one in Wikipathways pathway. The more fine-grained GO terms were mainly summarized into different functional categories: transcriptional and post-transcriptional regulation, synapse, endocytic membrane traffic through the endosomal system, signaling transduction, immune process, multi-organism process, protein catabolic metabolism, and cell adhesion. The shared pathways were roughly classified into three categories: immune system; cancer (NSCLC and glioma); and signal transduction pathways involving the cadherin signaling pathway, Wnt signaling pathway, G-protein signaling and downstream signaling mediated by phosphoinositides (PIPs). The majority of these common pathways linked to both AD and IS were supported by convincing evidence from the literature. In conclusion, our findings contribute to a better understanding of common biological mechanisms underlying AD and IS and serve as a guide to direct future research.

## Introduction

Alzheimer’s disease (AD) is the most common cause of dementia in the elderly. It is characterized pathologically by extracellular deposits of amyloid-β peptide (Aβ) and intracellular neurofibrillary tangles (NFTs) containing tau protein (Cuyvers and Sleegers, 2016). Ischemic stroke (IS) is a major health-threatening cerebrovascular disease with severe complications, such as post-stroke infection, physical disability, and cognitive deficits (Donnan et al., 2008). These two neurological diseases have immense socioeconomic impact worldwide (Norrving and Kissela, 2013; Alzheimer’sAssociation, 2016).

Mounting evidence suggests that there is a potential correspondence between AD and IS. Firstly, epidemiological studies have revealed that AD is a contributing factor to the development of IS (Chi et al., 2013; Tolppanen et al., 2013), and vice versa (Gamaldo et al., 2006). Secondly, AD and IS have common risk factors (e.g., hypertension, diabetes, obesity, and hyperlipidemia) (de Bruijn and Ikram, 2014; Boehme et al., 2017). Thirdly, recent evidence has indicated that brain ischemia can promote the development of AD by inducing β-/γ-secretase-mediated Aβ accumulation and tau protein gene alterations (Salminen et al., 2017; Pluta et al., 2018). Fourth, neuroinflammation elicited by the immune system is thought to play essential roles in the development and progression of both AD and IS (Liu Y.H. et al., 2013; Anrather and Iadecola, 2016). Lastly, research shows that abnormal tau protein also plays crucial roles in IS (Tuo et al., 2017). Together, these findings support the hypothesis that shared genetic risk factors link AD and IS at the epidemiological and pathophysiological levels.

Recent genome-wide association studies (GWASs) have provided profound insights into the complex genetic architecture of AD (Shen et al., 2015; Li et al., 2016; Liu et al., 2016; Zhang et al., 2016; Jun et al., 2017; Sims et al., 2017) and IS (Traylor et al., 2012; Williams et al., 2013; Kilarski et al., 2014; Malik et al., 2016; Network et al., 2016; Crawford et al., 2018). Most recently, 29 risk loci for AD (Jansen et al., 2018) and 28 loci associated with IS and its subtypes [i.e., large vessel disease (LVD), cardioembolic stroke (CE), and small vessel disease (SVD)] (Malik et al., 2018) have been identified. Despite these findings, no significant genetic variants shared by AD and IS or its subtypes were discovered. From the perspective of pathways, however, four gene ontology (GO) categories jointly associated with AD and SVD were identified (Traylor et al., 2016). Moreover, single-disease pathway analysis demonstrates that AD and IS share common pathways that involve natural killer (NK) cells, i.e., NK cell mediated cytotoxicity in AD (Lambert et al., 2010; Liu et al., 2014; Chen et al., 2016; Jiang et al., 2017) and NK cell signaling in IS (Malik et al., 2016). These findings imply that the link between AD and IS may have to do with shared genetic signals at the pathway level.

Complex diseases like AD and IS are mostly driven by the joint interactions of associated genes affected by large proportions of SNPs well below genome-wide significance, and the crosstalk of regulatory pathways (Furlong, 2013). Based on this concept, pathway-based analysis has been an effective strategy to investigate the potential mechanisms of complex disease (Luo et al., 2010; Jin et al., 2014), thus is widely used to unravel either single disease etiology or pleiotropism of clinically distinct diseases (Lesnick et al., 2007; Liu G. et al., 2012; Liu Y. et al., 2013; Liu et al., 2014; Bao et al., 2015; Xiang et al., 2015). Taken together, these considerations prompted us to perform pathway analyses using AD and IS GWAS data to further dissect their common molecular mechanisms.

## Materials and Methods

### Samples

We obtained AD GWAS summary data from the International Genomics of Alzheimer’s Project (IGAP). IGAP performed a large, two-stage joint association analysis of AD on individuals of European descent (Lambert et al., 2013). In stage 1 (discovery stage), 17,008 AD cases and 37,154 controls from four previously published GWAS samples were included. These were the European Alzheimer’s disease Initiative (EADI), the Alzheimer Disease Genetics Consortium (ADGC), the Cohorts for Heart and Aging Research in Genomic Epidemiology Consortium (CHARGE), and the Genetic and Environmental Risk in AD Consortium (GERAD). After quality control procedures, 7,055,881 SNPs remained for further analysis. In stage 2 (replication stage), the top SNPs with a *P*-value less than 1E-3 in stage 1 were selected for replication in an independent sample (8,572 cases and 11,312 controls). Finally, the results of the two stages were combined in a meta-analysis in order to identify loci reaching genome-wide significance (*P* < 5.00E-08). In the present study, we used the GWAS summary data from stage 1.

We selected the IS GWAS summary results from the METASTROKE collaboration published by Malik et al (Malik et al., 2016). In the discovery stage, METASTROKE conducted a meta-analysis of 12 case-control GWAS comprising 10,307 Caucasian IS cases and 19,326 Caucasian controls, producing quality-controlled 8.3 million SNPs for further analysis. In the replication stage, the top SNPs (*P* < 1.00E-05) in the discovery meta-analysis were selected for replication in three independent samples consisting of Caucasian (13,435 cases and 29,269 controls) and South Asian (2,385 cases and 5,193 controls) samples. Ultimately, a transethnic meta-analysis combining the two stages was conducted. Here, we used the IS GWAS summary statistics from the discovery phase.

### Statistical Analyses

Firstly, we computed gene-level association statistics through gene-based association tests. Next, gene-based pathway analysis was performed to identify pathways shared between AD and IS.

#### Gene-Based Testing for AD and IS GWAS Datasets

We performed gene-level analysis implemented in the versatile gene-based association study software (VEGAS). By incorporating the effects of a full set of markers within a gene and correcting for linkage disequilibrium (LD) between SNPs, we were able to calculate gene-based *P*-values. Compared with other gene-based testing approaches like PLINK, a set-based test based on permutations (Purcell et al., 2007), VEGAS is much more computationally efficient by using simulations from multivariate normal distributions (Liu et al., 2010). In addition, because VEGAS only requires SNP rs IDs and *P-*values, it is particularly appropriate for GWAS summary results, in which individual genotyped information is not available. We used VEGAS2, a recently updated version of VEGAS that uses LD estimates from the 1000 Genomes Project phase I panel (Mishra and Macgregor, 2015). We chose the following defaults to define the gene boundaries for locating SNPs in VEGAS2: “SNPs within a gene plus SNPs outside of the gene with *r*^2^ > 0.8 with SNPs in the gene” (Mishra and Macgregor, 2015). By using this default setting, we were able to capture the effects of regulatory SNPs and minimize non-specificity caused by large boundaries (i.e., ±50 kb) (Mishra and Macgregor, 2015).

Recently, Boyle et al. proposed the hypothesis that most complex traits are largely driven by the vast majority of peripheral genes with smaller effects by propagating through regulatory networks to core genes (Boyle et al., 2017). As such, genes in AD and IS with *P* < 0.05, rather than with stringent threshold like gene-wide statistical significance, were selected for subsequent pathway analysis.

#### Pathway-Based Analysis for Shared Disease Pathways

It is likely that genes of AD and IS are involved in one pathway, but in different proportions, such as in the upstream and downstream of the pathway, respectively. Hence, we performed pathway analysis for AD and IS separately by integrating genes for each disease from VEGAS2 (*P* < 0.05) into known functional annotations using WebGestalt (Wang et al., 2017). Next, their respective enrichment results were overlapped to identify shared pathways jointly associated with AD and IS.

The latest version of WebGestalt 2017 included genetic pathways from GO annotations (Ashburner et al., 2000), KEGG (Kanehisa et al., 2004), PANTHER (Mi et al., 2016), Reactome (Fabregat et al., 2016), and WikiPathways (Pico et al., 2008). We chose over-representation analysis (ORA), a hypergeometric test, to identify the enrichment of the disease-related genes among all the genes in a given pathway. The *P*-value for observing at least **m** disease-related genes in a given pathway can be calculated as follows:

$$P=1-\sum_{i=0}^{m}\frac{\left(\begin{array}{c} n\\ i \end{array}\right)\left(\begin{array}{c} N-n\\ M-i \end{array}\right)}{\left(\begin{array}{c} N\\ M \end{array}\right)}$$

where M is the total number of genes of interest that are associated with a given disease, N is the number of reference genes, and n is the number of genes in the pathway. The smaller the *P*-value is, the more likely the list of disease-related genes of interest will be overrepresented in this pathway. To avoid testing overly narrow or broad pathways, which could result in some false positives, we chose pathways comprising at least 20 and at most 300 genes for analysis (Jiang et al., 2017). The threshold of statistical significance for a pathway associated with AD and IS was *P* < 0.05 in both diseases.

#### Meta-Analysis Using Fisher’s Method

Here, Fisher’s method was used to combine the *P*-values of each shared pathway in AD and IS into one test statistic. For a given pathway, the Fisher equation for the statistic is:

$$x^{2}=-2\sum_{i=1}^{k}\ln(P_{i})$$

where *P*_i_ is the *P*-value of the pathway in the i_th_ study and k is the number of total studies. x^2^ follows chi-square distribution with 2k degrees of freedom (Begum et al., 2012).

## Results

### Gene-Based Testing for AD and IS GWAS Datasets and Validation of VEGAS2

We identified 1915 AD genes and 1288 IS genes with *P* < 0.05. More detailed results are provided in **Supplementary Table S1**. To verify the reliability of the VEGAS2 gene-based testing approach, we compared our gene-based association results of AD with previously established AD risk loci identified through conventional GWAS approaches. We replicated 15 genes (*APOE, TOMM40, APOC1, EXOC3L2, PVRL2, CR1, HLA-DRB6, EPHA1, CLU, PICALM, ABCA7, CD33, MS4A2, MS4A6A, BIN1*) with gene-wide significance (*P* < 2.35E-6, Bonferroni corrected for 21,244 genes). These genes belong to 12 established risk loci for AD (i.e., *APOE/TOMM40/APOC1* locus; *EXOC3L2/BLOC1S3/MARK4* locus; *PVRL2; CR1; HLA-DRB5-DRB1* region; *EPHA1; CLU; PICALM; ABCA7; CD33; MS4A* locus*; BIN1*). In addition, we replicated 11 genes (*SORL1, CD2AP, MS4A6E, MS4A4A, MS4A3, HLA-DQB1, HLA-DRB1, HLA-DQA1, PTK2B, CELF1*, and *SLC24A4*) with suggestive association (*P* < 1.00E-04); these belong to seven known significant loci (*SORL1, CD2AP, MS4A* locus, *HLA-DRB5-DRB1* region, *PTK2B, CELF1*, and *SLC24A4*). Thus, the replication of these established findings shows that VEGAS2 can be trusted as a validated tool for our subsequent pathway analysis.

### Pathway-Based Analysis for Shared Pathways Between AD and IS

Using the 1915 AD genes and 1288 IS genes having a *P* < 0.05, pathway analysis for AD and IS were separately conducted followed by overlapping their respective significant enrichment results.

**FIGURE 1:**
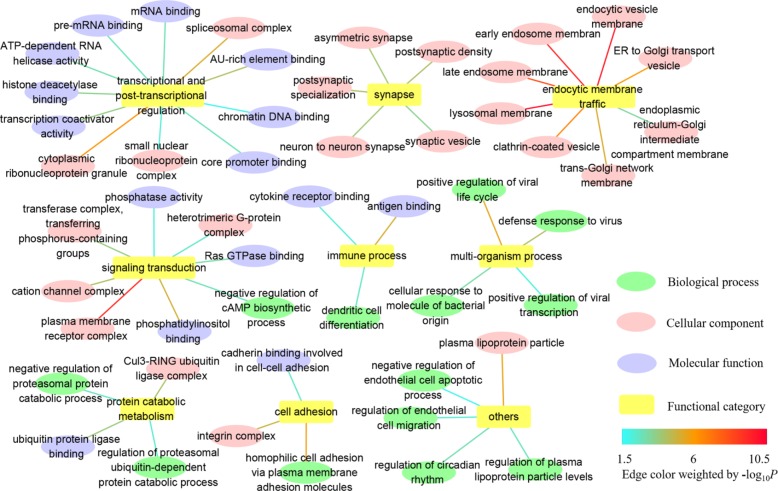
Summarized GO annotation results shared by AD and IS. The complete GO annotation results shared by AD and IS are summarized and reorganized here, with only more fine-grained terms labeled and classified into different functional categories: transcriptional and post-transcriptional regulation, synapse, endocytic membrane traffic, signaling transduction, immune process, multi-organism process, protein catabolic metabolism, cell adhesion, and others, which are shown in nodes. The relevant GO terms of biological process, cellular component, and molecular function are shown in targets. The threshold for statistical significance for a GO term is *P* < 0.05 in both AD and IS. The edges connecting the nodes and targets are attributed by the value of –log_10_ *P*.

In GO, we identified 179 common GO categories associated with both AD and IS: 56 biological processes, 95 cellular components, and 28 molecular functions (**Supplementary Table S2**). Here, we paid more attention to the more fine-grained GO terms and weeded out their ancestors to solve the problem of redundancy. Taken as a whole, the remaining GO terms of biological process, cellular component and molecular function mainly fall into categories listed below: transcriptional and post-transcriptional regulation, synapse, endocytic membrane traffic through the endosomal system, signaling transduction, immune process, multi-organism process, protein catabolic metabolism, cell adhesion, and others (**Figure 1** and **Supplementary Table S2**). The detailed information about complete GO annotation results and summarized GO annotation results as well as their relevant GO terms is displayed in **Supplementary Table S2**.

In KEGG, we identified six significant pathways shared by AD and IS: NK cell mediated cytotoxicity [hsa04650], Toll-like receptor signaling pathway [hsa04620], non-small cell lung cancer [hsa05223], glioma [hsa05214], phospholipase D signaling pathway [hsa04072], and hepatitis B [hsa05161]. Besides these, we also identified 2 shared significant PANTHER pathways between AD and IS: cadherin signaling pathway [P00012] and Wnt signaling pathway [P00057]. Four significant Reactome pathways shared by AD and IS were immunoregulatory interactions between a lymphoid and a non-lymphoid cell [R-HSA-198933], synthesis of PIPs at the plasma membrane [R-HSA-1660499], PI metabolism [R-HSA-1483255] and cooperation of PDCL (PhLP1) and TRiC/CCT in G-protein beta folding [R-HSA-6814122]. In the WikiPathways dataset, one significant pathway was identified: signaling pathways in glioblastoma [WP2261]. The detailed results are described in **Table 1**.

**Table 1:** Shared biological pathways between AD and IS.

Pathway ID	Pathway name	AD					IS					*P*_combined_
		C	O	E	R	*P-*value	C	O	E	R	*P-*value
**KEGG**		
hsa05214	Glioma	66	11	5.19	2.12	1.82E-02	66	8	3.38	2.37	3.62E-03	6.59E-05
hsa04620	Toll-like receptor signaling pathway	106	14	8.33	1.68	1.09E-02	106	10	5.42	1.84	7.15E-03	7.79E-05
hsa05223	Non-small cell lung cancer	56	10	4.4	2.27	1.30E-02	56	8	2.86	2.79	1.86E-02	2.42E-04
hsa04650	Natural killer cell mediated cytotoxicity	135	18	10.61	1.7	8.60E-03	135	15	6.91	2.17	3.25E-02	2.80E-04
hsa04072	Phospholipase D signaling pathway	144	20	11.32	1.77	3.68E-02	144	13	7.37	1.76	3.57E-02	1.31E-03
hsa05161	Hepatitis B	146	18	11.48	1.57	3.72E-02	146	13	7.47	1.74	4.40E-02	1.64E-03
**PANTHER**		
P00012	Cadherin signaling pathway	153	24	13.30	1.80	2.46E-03	153	21	8.94	2.35	1.32E-04	3.25E-07
P00057	Wnt signaling pathway	294	35	25.57	1.37	2.71E-02	294	30	17.17	1.75	1.07E-03	2.90E-05
**REACTOME**		
R-HSA-198933	Immunoregulatory interactions between a Lymphoid and a non-Lymphoid cell	132	20	10.23	1.96	2.79E-03	132	14	6.54	2.14	5.75E-03	1.60E-05
R-HSA-1660499	Synthesis of PIPs at the plasma membrane	49	8	3.8	2.11	3.32E-02	49	8	2.43	3.29	2.58E-03	8.57E-05
R-HSA-6814122	Cooperation of PDCL (PhLP1) and TRiC/CCT in G-protein beta folding	43	8	3.33	2.4	1.60E-02	43	6	2.13	2.81	1.85E-02	2.96E-04
R-HSA-1483255	PI metabolism	78	11	6.04	1.82	3.72E-02	78	9	3.87	2.33	1.47E-02	5.47E-04
**WIKIPATHWAYS**		
WP2261	Signaling pathways in glioblastoma	83	12	6.81	1.76	3.68E-02	83	10	4.65	2.15	1.67E-02	6.15E-04

*C, the number of reference genes in the category; O, the number of genes in the list of disease-related genes of interest and also in the category; E, the expected number in a category; R, the ratio of enrichment. The threshold of statistical significance for a pathway associated with AD and IS was P < 0.05*.

## Discussion

Large-scale GWAS of AD and IS have identified a set of risk loi with genome-wide significance and provided deep insights into the genetics of AD and IS (Cuyvers and Sleegers, 2016; Boehme et al., 2017). Despite these discoveries, a large proportion of heritability remains underappreciated (Zhang et al., 2013; Markus and Bevan, 2014). To date, no shared genetic determinants jointly associated with AD and IS have been revealed. However, AD and IS have common biological pathways that may account for their similarities at the epidemiological, neuropathological, and molecular levels. Indeed, our pathway-based association tests using large-scale GWAS summary datasets for AD and IS prove the genetic links between these two diseases at the pathway level.

In order to fully understand the biological features shared by AD and IS, we included in our analyses different functional annotation databases (i.e., GO annotation, KEGG, PANHTER, Reactome, and Wikipathways). GO annotation sets represent the most comprehensive spectrum of functional categories but are mainly computationally inferred, with less than 1% being experimentally confirmed (Ashburner et al., 2000). In contrast, KEGG, PANTHER, and Reactome pathway databases are manually curated from the literature based on experimentally validated evidence (Kanehisa et al., 2004; Fabregat et al., 2016; Mi et al., 2016). Compared to the centrally curated databases above, open community-based Wikipathways allows for broader exploration into the entire biological spectrum (Pico et al., 2008). Altogether, we uncovered 179 GO annotation terms, 6 shared KEGG pathways, 2 PANTHER pathways, 4 Reactome pathways, and one pathway in Wikipathways jointly associated with AD and IS.

### Shared GO Annotations

The GO annotation provides a knowledge base for understanding the roles of genes and proteins in cells. Complete GO schema generates a huge number of annotation terms with parent–child relationships, which are non-independently arranged as a directed acyclic graph (DAG). To assist in interpretation, we tried to focus on more fine-grained terms, allowing more specific biological mechanisms to be dissected. These descendants turned out to be relevant to the following functional categories: transcriptional and post-transcriptional regulation, synapse, endocytic membrane traffic through the endosomal system, signaling transduction, immune process, multi-organism process, protein catabolic metabolism, cell adhesion and others (**Figure 1**). In particular, dysfunction of endocytic membrane dynamics plays a key role in AD pathogenesis by enhancing the processing of amyloid precursor protein (APP) into Aβ (Wu and Yao, 2009; Rajendran and Annaert, 2012). However, the relationship between the endosomal system and IS remains unknown and warrants further extensive investigation.

### Shared KEGG Pathways

Together with one Reactome pathway [R-HSA-198933 immunoregulatory interactions between a lymphoid and a non-lymphoid cell], two KEGG pathways are related to the immune system (i.e., Toll-like receptor signaling pathway [hsa04620] and NK cell mediated cytotoxicity [hsa04650]). Toll-like receptor signaling pathway has also been reported to be associated with AD (Liu et al., 2014). It is now widely accepted that Toll-like receptors (TLRs) play essential roles in Aβ-induced microglial inflammatory activation in AD (Liu S. et al., 2012). The persistent inflammatory stimuli fueled by activated microglia in turn leads to a disturbance in microglial clearance of Aβ, eventually causing neuronal degeneration (Heneka et al., 2015). Following IS, damage-associated molecular patterns (DAMPs) released from the intracellular compartment of dying and dead cells activate microglia and perivascular macrophages by pattern recognition receptors (PRRs), especially TLRs. The triggering cascades of innate immunity further exacerbate stroke lesions in the acute phase of IS (Iadecola and Anrather, 2011).

Natural killer cell mediated cytotoxicity has been reported by previous GWAS and brain expression datasets of AD (Lambert et al., 2010; Liu et al., 2014; Chen et al., 2016; Jiang et al., 2017). The association for IS and NK cell signaling was also observed (Malik et al., 2016), and supported by evidence that infiltrating NK cells exacerbate ischemia lesions and increase neuronal death either in experimental IS models or in human IS (Gan et al., 2014). The inflammatory responses in both AD and IS are initiated locally within the central nervous system (CNS) under aberrant local conditions, and presumably elicited by the innate immune system. Therefore, the similarity of their pathophysiological processes may provide a reasonable explanation for our findings that AD and IS share common biological pathways involved in the immune system.

Another two KEGG pathways are related to cancer (i.e., glioma [hsa05214] and non-small cell lung cancer [hsa05223]). Convincing evidence suggests an inverse relationship and significant genetic overlap between AD and cancer (Roe et al., 2005; Driver et al., 2012; Musicco et al., 2013; Ou et al., 2013). For instance, the cellular behavior of proliferative cells in cancer is essentially the opposite of the processes leading to degenerating neurons in AD (Behrens et al., 2009; Driver and Lu, 2010; Driver, 2014). Bioinformatics analyses has unraveled shared genes and signaling pathways connecting AD and glioblastoma (Liu T. et al., 2013). The one significant pathway we found in the Wikipathways dataset (signaling pathways in glioblastoma [WP2261]) also supports this linkage. Though some illustrative cases suggested that glioblastoma should be considered in the etiology of acute IS (Pina et al., 2014; Lasocki and Gaillard, 2016), little is known about the relationship between IS and glioma or other prevalent cancers.

The biological significance of the phospholipase D (PLD) signaling pathway [hsa04072] has been suggested for both AD and IS (Oliveira and Di Paolo, 2010; Stegner et al., 2013; Frohman, 2015). Hepatitis B pathway [hsa05161] has already been revealed in hippocampal CA1 region of AD patients (Mastroeni et al., 2018). Epidemiology studies suggested that hepatitis B virus (HBV) infection was associated with decreased risk of IS, although the underlying mechanism was unclear (Sung et al., 2007; Kuo et al., 2017). Part of the GO terms we deciphered, i.e., positive regulation of viral transcription [GO:0050434], positive regulation of viral life cycle [GO:1903902] and defense response to virus [GO:0051607] appeared to support this linkage.

### Shared PANTHER Pathways

In the present study, we uncovered 2 significant PANTHER pathways shared by AD and IS (cadherin signaling pathway [P00012] and Wnt signaling pathway [P00057]). Similar to cadherin signaling pathway, cadherin binding involved in cell-cell adhesion [GO:0098641] was implied in GO. Both enriched PANTHER pathways primarily arose from the *Pcdhα* gene cluster (*Pcdhα*) shared by AD and IS in our VEGAS2 gene-based analysis. Protocadherins (Pcdhs) constitute the largest group of the cadherin superfamily of cell-adhesion molecules. Pcdhs are predominantly expressed in the CNS (i.e., neurons, astrocytes, pericytes, choroid plexus epithelial cells, and brain microvascular endothelial cells), and are crucial for neuronal survival, neural circuit assembly, and maintenance of the blood-brain barrier (BBB) (Takeichi, 2007; Dilling et al., 2017). Moreover, the interactions between Pcdhs and Wnt signaling pathway have recently been uncovered (Mah and Weiner, 2017). Wnt signaling pathway plays critical roles in neurogenesis and synaptic transmission and plasticity, whereby its deregulation shows significant linkage to the development of AD (Caruso et al., 2006; De Ferrari et al., 2007; Inestrosa and Varela-Nallar, 2014). For IS, experimental evidence has demonstrated that Wnt signaling activation may exert effective neuroprotection by providing an appropriate compensatory microenvironment for neurogenesis or neuronal survival in focal ischemic injury (Shruster et al., 2012; Wu et al., 2015).

### Shared Reactome Pathways

Two shared Reactome pathways are PI (phosphatidylinositol) metabolism [R-HSA-1483255] and synthesis of PIPs (phosphoinositides) at the plasma membrane [R-HSA-1660499], which are connected by a parent–child relationship. Similarly, phosphatidylinositol binding [GO:0035091] was also identified in GO. PIPs consist of seven diverse phosphorylated forms of phosphatidylinositol. Among these, PI(4,5)P_2_ and PI(3,4,5)P_3_ occur in relatively high abundance at the plasma membrane (Di Paolo and De Camilli, 2006; Riehle et al., 2013). PI(4,5)P_2_ primarily serves as a pool for the formation of intracellular second messengers—inositol 1,4,5-trisphosphate (IP_3_), diacylglycerol (DAG), and PI(3,4,5)P_3_— to mediate downstream signal cascades [e.g., Ca^2+^ response, PKC activation and PI(3,4,5)P_3_/Akt/mTOR pathway] from plasma membrane receptors like GPCRs (Riehle et al., 2013). PIPs play important roles in the nervous system by regulating lipid signaling, receptor signaling and membrane dynamics (e.g., endocytic membrane traffic). Thus, deregulation of these pathways has been implicated in neurological diseases including AD and IS (Waugh, 2015).

Another significant Reactome pathway shared by AD and IS is cooperation of PDCL (PhLP1) and TRiC/CCT in G-protein beta folding [R-HSA-6814122]. Recent studies reveal the interplay of the protein-folding chaperone CCT (also called TRiC) and its co-chaperone PhLP1 in G-protein βγ (Gβγ) dimer formation, which is a particularly critical step in G-protein signaling (Plimpton et al., 2015). Comparably, heterotrimeric G-protein complex [GO:0005834] was also identified in GO. In AD, G-protein signal transduction pathways mediated by G protein-coupled receptors (GPCRs) are strongly related to α-/β-/γ-secretase-mediated APP processing through the endosomal system (Teng et al., 2010; Thathiah and De Strooper, 2011; Heese, 2013).

In summary, we discovered several shared significant pathways jointly associated with AD and IS, which can roughly be classified into three categories: immune system, cancer (NSCLC and glioma), and signal transduction pathways. The latter includes cadherin signaling pathway, Wnt signaling pathway, G-protein signaling, and downstream signaling mediated by PIPs. Convincing evidence in the literature corroborates the majority of these shared pathways.

Despite these interesting and consequential findings, our study had some limitations. Firstly, we were able to analyze only the overall IS summary data. However, IS is genetically heterogeneous, as nearly all its risk loci are specific to individual subtypes. Secondly, the input lists of significant genes for pathway analysis were selected by setting an arbitrary threshold (*P* < 0.05), which might lead to information loss of some marginally less significant genes (e.g., *P* = 0.051). Lastly, the statistical model we used for pathway analysis was ORA, a hypergeometric test, which treats each gene equally and does not take into account interdependencies between genes in a pathway. However, the significance of each gene (*P*-value) can be informative in assigning different weights to input genes in the pathway analysis. In the future, we aim to conduct more robust statistical analysis using multiple pathway analysis methods and multiple datasets to replicate and expand our findings.

## Conclusion

Collectively, in our gene-based tests and comprehensive pathway analyses of AD and IS summary GWAS datasets, we discovered several shared novel functional pathways linking AD and IS. By providing an in-depth investigation here of the shared biological mechanism associated with these two neurological disorders, our findings may advance the current understanding of the biology of AD and IS and guide future research on AD or IS in new directions.

## Author Contributions

PC accomplished by the research. JH applied for the GWAS data of AD and IS. PC completed the data analysis and drafted the manuscript. XM, HL, and WL were responsible for manuscript revision. The final version was approved for submission by all listed authors.

## Conflict of Interest Statement

The authors declare that the research was conducted in the absence of any commercial or financial relationships that could be construed as a potential conflict of interest.

## Supplementary Material

TABLE S1 Gene-based testing results for the GWAS datasets of AD and IS.

TABLE S2 GO annotation results shared by AD and IS.
